# Describing Chronic Malaria Infections

**DOI:** 10.4269/ajtmh.19-0085a

**Published:** 2019-04

**Authors:** Andrew W. Taylor-Robinson

**Affiliations:** School of Health, Medical and Applied Sciences; Central Queensland University; Brisbane, Australia; E-mail: a.taylor-robinson@cqu.edu.au

Dear Sir,

I read with interest the recent thought-provoking article by Dennis Shanks.^1^ This discusses the state of anti-disease resistance that may eventually be achieved by a person if he/she is repeatedly exposed to infection with *Plasmodium falciparum*, assuming that no infectious episode has a fatal outcome. This “steady state” between parasite dispersal and host survival can be observed in those adult residents of *P. falciparum*–endemic areas who have not succumbed to exposure during childhood. The author makes a compelling case for using the term “malaria-tolerant” in preference to “malaria-immune,” which is found far more frequently in the published literature. The reason for this is that such individuals often harbor blood-stage parasites below a threshold of parasitemia required for them to show clinical manifestations of malarial infection, and thus they are apparently tolerant to the residual but persistent presence of parasites in their peripheral circulation. Hence, their naturally acquired protective immune response, a form of premunition, reduces infection for the most part to subclinical levels, but does not eradicate all parasites. It is likely that these asymptomatic yet chronic carriers of malaria parasites provide a reservoir of infection for local transmission if and when an *Anopheles* mosquito takes a blood meal from them, especially if this bite coincides with a recrudescence of patent infection.

Shanks is being pedantic, in the very best sense of the word, in choosing “tolerant” over “immune” in relation to chronic *P. falciparum* infections. Continuing this vein of academic pedantry, I wish to raise a point that some may consider to be trivial in the context of the important underlying message that is conveyed by this article. Nevertheless, in my opinion, there is an ambiguity that should be clarified. The first three sentences read as follows: “Adults who have not grown up in a malaria-endemic area may experience severe malaria soon after entering a malarious area. Such mortality is usually limited to a short period of time (months), after which they are thought to be ‘immune’. Such anti-disease immunity may be more accurately considered as tolerance.” Drawing attention to the second sentence, to my mind if mortality is experienced by a person, the only reason they will be rendered immune to further malarial infection is because they will in fact be dead. Although it may be argued, tongue planted firmly in cheek, that host mortality is the ultimate expression of non-protective immunity, it is not a connotation that I think the author means to convey. As such, Shanks appears inadvertently to confuse mortality with morbidity, or perhaps the duration of risk of mortality. It would be better to write the following, or a similar form of words: “Such potentially life-threatening clinical manifestations are usually limited to a short period of time (months), after which, if the person survives, they are thought to be ‘immune’.” Of course, given the tenet of his argument, the author may prefer “Such potentially life-threatening clinical manifestations are usually limited to a short period of time (months), after which, if the person survives, they are thought to be ‘tolerant’.”
